# Author Correction: Comparing storm resolving models and climates via unsupervised machine learning

**DOI:** 10.1038/s41598-024-57767-8

**Published:** 2024-03-28

**Authors:** Griffin Mooers, Mike Pritchard, Tom Beucler, Prakhar Srivastava, Harshini Mangipudi, Liran Peng, Pierre Gentine, Stephan Mandt

**Affiliations:** 1 Department of Earth System Science, University of California at Irvine, Irvine, CA 92697 USA; 2 NVIDIA, Santa Clara, CA 95050 USA; 3 Institute of Earth Surface Dynamics, University of Lausanne, 1015 Lausanne, Switzerland; 4 Department of Computer Science, University of California at Irvine, Irvine, CA 92617 USA; 5 Department of Earth and Environmental Engineering, Columbia University, New York, NY 10027 USA

Correction to: *Scientific Reports* 10.1038/s41598-023-49455-w, published online 15 December 2023

The original version of this Article omitted an affiliation for Mike Pritchard. The correct affiliations are listed below.

Department of Earth System Science, University of California at Irvine, Irvine, CA 92697, USA.

NVIDIA, Santa Clara, CA 95050, USA.

The original Article has been corrected.

