# Supplementary material for: Comparing storm resolving models and climates via unsupervised machine learning
Source: Sci Rep. 2023 Dec 15;13:22365. doi: 10.1038/s41598-023-49455-w (PMC10724240; doi:10.1038/s41598-023-49455-w)
Supplement: Supplementary file 1 — Supplementary Information. [file 41598_2023_49455_MOESM1_ESM.pdf]

Supplementary Information for

Comparing Storm Resolving Models and Climates via Unsupervised Machine Learning

Griffin Mooers, Mike Pritchard, Tom Beucler, Prakhar Srivastava, Harshini Mangipudi, Liran Peng, Pierre Gentine, Stephan Mandt

Griffin Mooers
E-mail: gmooer96@gmail.com

This PDF file includes:

Supplementary text
Figs. S1 to S13 (not allowed for Brief Reports)
Legends for Movies S1 to S6
SI References

Other supplementary materials for this manuscript include the following:

Movies S1 to S6

Supporting Information Text

A. Robustness of Clustering. To confirm the robustness of these clusters, we perform a hyper-parameter sweep over the clustering routine type (K++ or true K-Means) and the number of initializations. From one hundred trials, we observe a combination of the more modern K++ algorithm (2) and sufficient initializations (ten) yields three reproducible clusters (Figure S1)

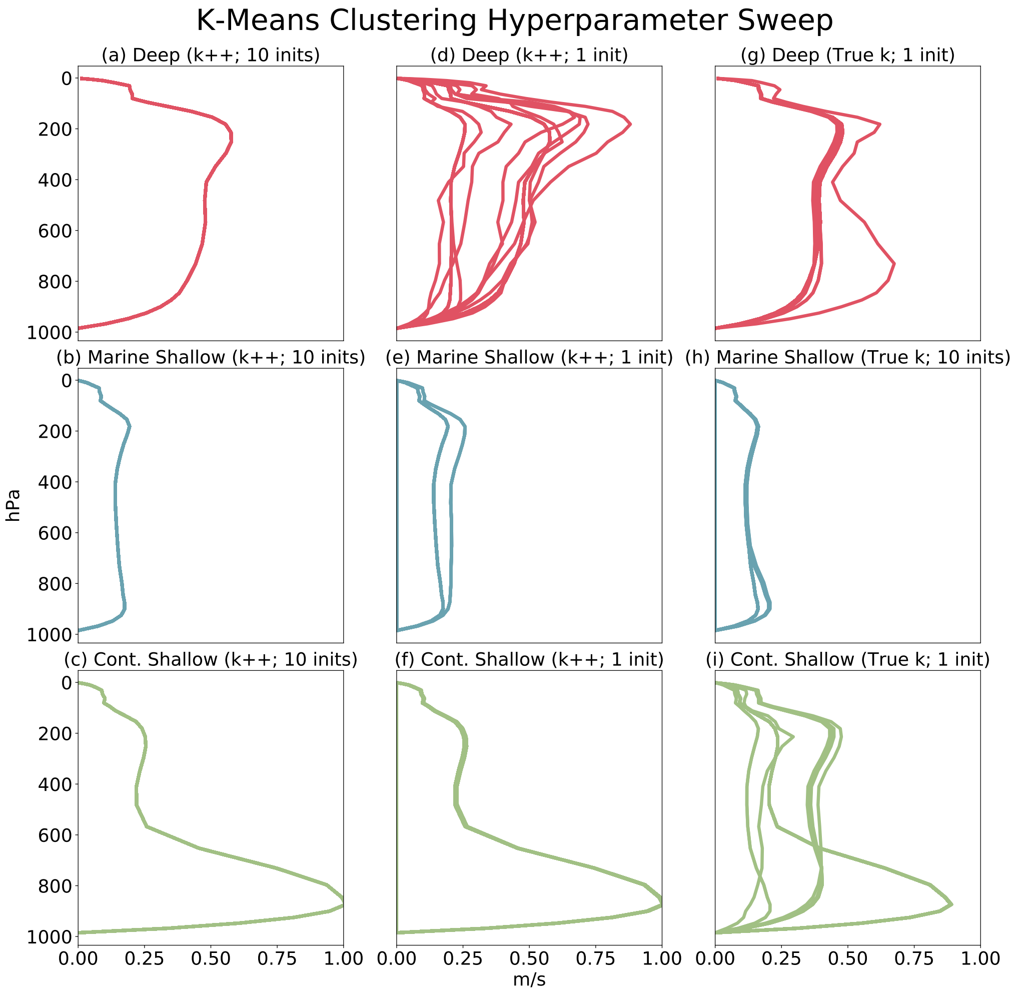

Fig. S1. Our hyperparameter sweep for the K-means clustering algorithm. In all cases we set $K = 3$, but sweep over algorithm choice (K++ vs. true K-means) and number of initializations. Each panel displays the median vertical structure of a cluster. A smaller number of profiles indicates more robust clusters across 100 unique trials.

B. Baselines. Our data-driven inter-comparison approach for GSRMs incorporates two machine learning techniques: (1) Non-linear dimensionality reduction, and (2) K-Means clustering. To validate the efficacy of our workflow and in particular, the need for a *non-linear* dimensionality reduction, we conducted baseline experiments and ablations. Our findings strongly support the importance of representation learning (*non-linear* dimensionality reduction) for meaningful distributional comparisons. Additionally, we demonstrate the robustness of our clustering approach to different initializations and random seeds.

First, the robustness of our clustering approach is evidenced by distinct clusters of convection with recognizable physical properties (Figure 3 b-d) that are consistently observed (Figure S1). We can observe the separation of these physical properties when we colorize the latent space by established physical quantities such as intensity statistics or the geographic location of the convection sample.

Next, in order to assess the importance of non-linear dimensionality reduction, we compare our approach against the trivial baseline of clustering the full vertical velocity fields in the raw pixel space. However, even with reduced stochastic hyperparameter choices (ten unique initializations of the k++ algorithm), we find that reproducible clusters could not be achieved across 100 trials (Figure S2 a,b,c vs. g,h,i). We further strengthen our claim for this by exploring an alternative dimensionality reduction approaches. We employ Principal Component Analysis (PCA) to reduce the GSRM test data to the same size as the VAE latent representations, i.e., 1000 dimensions. We then perform clustering using the same procedure. Although the clusters obtained from PCA exhibit less variance compared to clustering in the raw pixel space, they are still not stable enough (Figure S2 d,e,f vs. a,b,c).

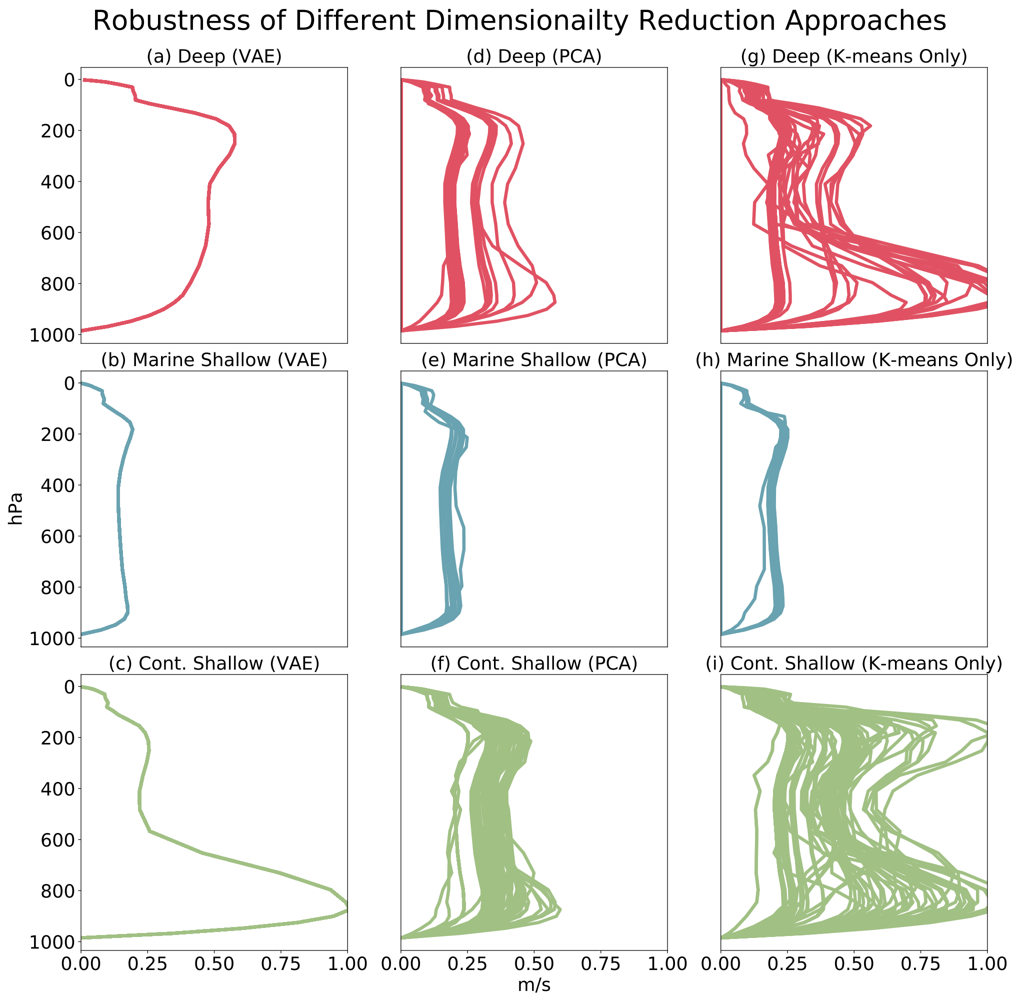

Fig. S2. K-means clustering performed on the latent representation of convection from a VAE encoder (a,b,c), clustering on convection after dimensionality reduction from PCA (d,e,f), and clustering directly on full resolution vertical velocity fields (g,h,i). In all cases we set $K = 3$, use the K++ algorithm, and ten initializations. Each panel displays the median vertical structure of a cluster. A smaller number of profiles indicates more robust clusters across different trials.

C. On the importance of convolutional filters. There is one piece of our VAE design that is especially important to highlight (for other information on VAE hyper-parameter testing please see (19)): the choice of a fully convolutional architecture. Convolutional Neural Networks (CNNs) have helped the machine learning community make great strides over the past several years (9) with the tasks of image classification (13), speech recognition (21), and object identification (22). The convolutional filters allow for feature extraction in high-dimensional images as well as the preservation and recognition of important spatial structures (9). Our previous empirical testing led us to believe that this convolutional structure is critical for the analysis of high resolution vertical velocity (19), and we now wish to more concretely demonstrate its importance.

The core task at hand is the non-linear dimensionality reduction of a high dimensional image (vertical velocity field), into a low dimensional, physically interpretable representation. We believe the convolutional structure we implement in our model allows for a more comprehensive analysis of each vertical field and for the extraction of the small scale structures that are essential to understanding the characteristics of convection, including details of both the horizontal and vertical structures.

We test the importance of fine-scale coherence in the vertical velocity fields for how the VAE organizes the GSRM data (In this case focusing on the SPCAM model). To that end, we generate a new test dataset drawn from the same simulation and randomly sampled with respect to time and geography). We now make two "clones" of this test data (and the original training data set). In the first instance, we shuffle the order of columns in the vertical velocity field image but do not perturb the vertical structure (Figure S3b), and in the second case, we scramble the information on each vertical level separately, disrupting both vertical and horizontal structure (Figure S3c).

Prior to this, we had a VAE which generated robust results for us – clusters that had distinct physical properties and were reproducible (Figure S2). Our theory is that without a coherent vertical structure, the results will be less robust. We repeat the VAE training and clustering procedure with two new VAEs, one trained on the partially scrambled data (Figure S3b) and one on the fully scrambled data (Figure S3c). Each, like the original VAE, has a cluster of deep convection in its latent space when it is clustered (Figure S3 (d-f), solid lines).

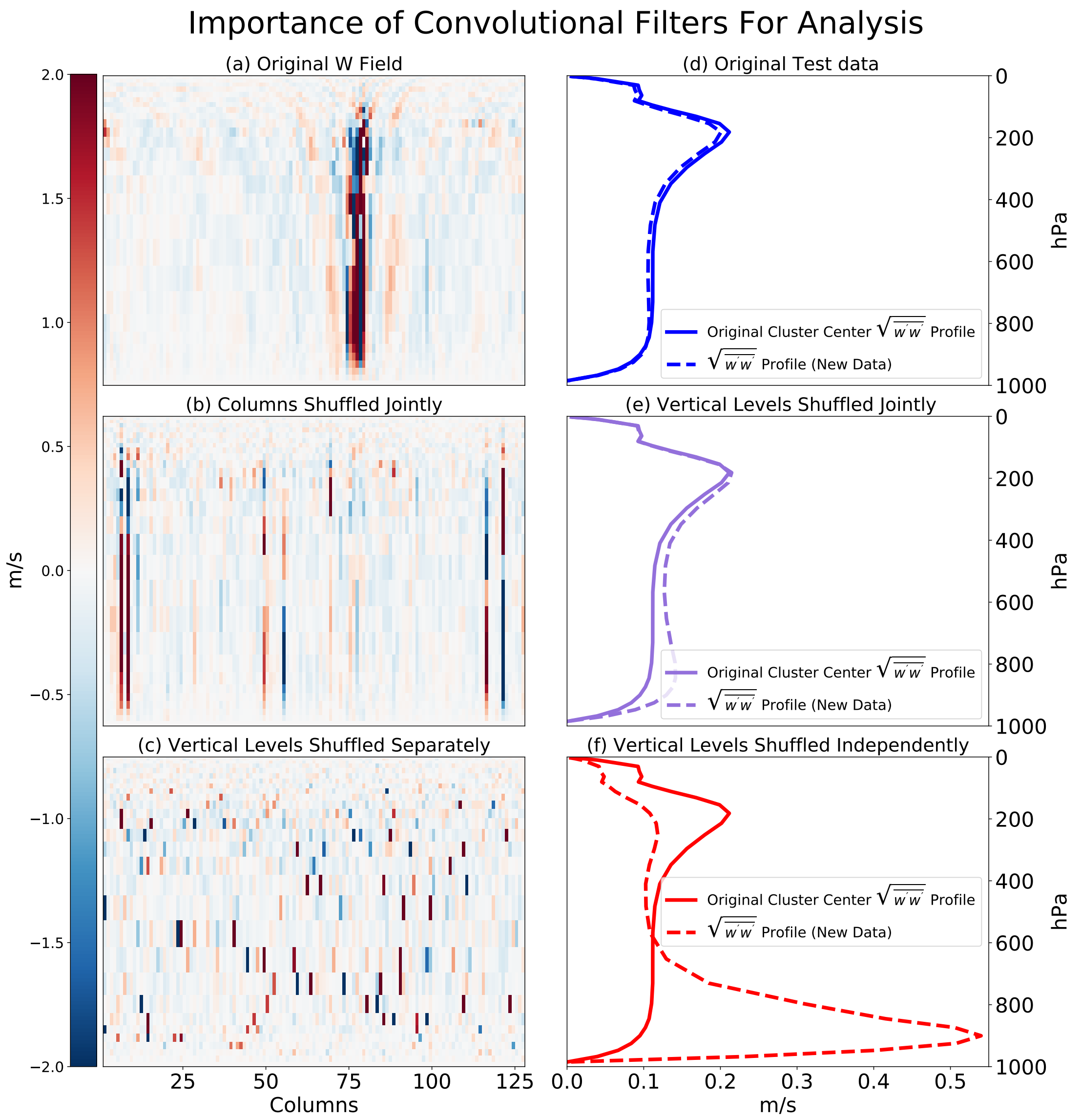

Fig. S3. Results of our investigation into the importance of convolutional filters utilizing the small-scale structures in our test data. Here we show an example of a standard vertical velocity field we use in our test data (a), the same field where we scramble the order of the columns (b), and the same field where we scramble the order of pixels at each vertical level separately. We show the results of shifts in the median profile of Deep Convection for VAEs trained on these three types of data, with cluster centers already initialized on one test dataset and applied to another. Greater shifts in the profiles (d-f) indicate greater shifts in the cluster physical characteristics between the two similar datasets and therefor less robustness and physical interpretability.

At first glance, this would suggest that this organization is not important to our results. However, differences emerge upon closer inspection of the robustness of the latent space. We introduce yet another test dataset to the VAEs, again randomly sampled and in both of the scrambled forms described above. Next, we initialize clustering on the latent representation of this test dataset with the cluster centers saved from the previous one.

If our clusters are robust and physically meaningful, we should see little movement in the cluster centers and the physical properties of each cluster. However, that is only the case when the test data is not scrambled – here the median deep convection profile of the cluster remains nearly identical ((Figure S3d) dotted vs. solid line). In both other cases, when the fine scale structure of the test dataset is perturbed, we lose this reproducibility. This is most obvious when the test data is scrambled both horizontally and vertically (Figure S3f), here the cluster center that was previously affiliated with deep convection shifted

almost entirely to a cluster composed of shallow convection. However, even when just the order of the columns is shuffled, we see a substantial cluster center shift as evidenced by shallow convection samples being incorporated into the regime of deep convection (Figure S3e.)

This lack of robustness from the perturbed test data highlights how essential it is to have a VAE capable of leveraging the fine-scale vertical velocity information for robust, physically interpretable latent representations of the GSRM data. The convolutional filters in our VAE are invaluable for just that.

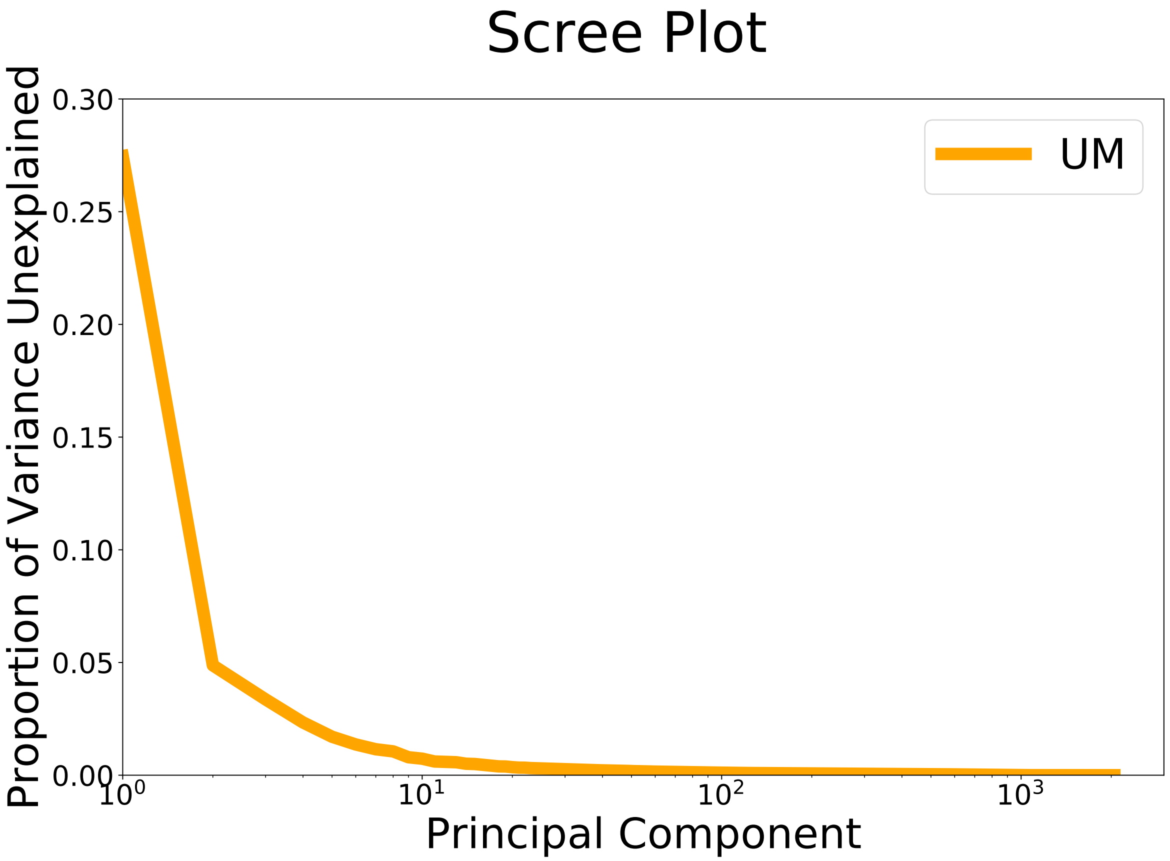

Fig. S4. The proportion of variance of the full 1000 dimensional encoding left unexplained as we project down from the full z vector to visualize the latent representation in 2D or 3D Space. We see the first three principal components are the most important for preserving the information from the latent vector.

D. Latent Space Projections. For much of our qualitative analysis, we rely on visual inspections of the latent space. Because of this, we need to verify sufficient information is preserved in these representations. Given the comparatively high dimension of $\mathbf{z}$, visualization is only possible with further compression. We rely on Principle Component Analysis (PCA) to linearly project $\mathbf{z}$ to just two (Figures 3, S4-6) or three (Movies S2-6) components for visualization. We acknowledge there will be a degree of information loss through this process. But we can quantify this compromise by examining a Scree (4) plot of the data. The Scree plot reveals how much of the variance of the full $\mathbf{z}$ vector can be explained by each principal component. Figure S4 suggests the first three, and in particular, the first two principal components are orders of magnitude more important than the others and thus we can project the latent representation down to a visible dimension and still conduct meaningful analysis.

E. A Common Encoder for Analysis. We elaborate on how we utilize a single VAE to facilitate a qualitative comparison among all nine high-resolution GSRM data simulations. Since direct quantification of differences between two high-dimensional DYAMOND GSRM simulations is challenging, we can instead treat the VAE as a density model to approximate and assess the qualitative differences. To that end, let $p_{\theta_A}(\mathbf{x}^A)$ be a generative model (VAE) trained on dataset A with learned parameters θ_A. We demonstrated above that we can leverage the encoder, $q_{\theta_A}(\mathbf{z}^A|\mathbf{x}^A)$, of the model to visualize the encoding of datatype A as $\mathbf{z}_A$ for novel dynamic analysis. But we now use the trained model encoder on another data, B, such that $q_{\theta_A}(\mathbf{z}^B|\mathbf{x}^B)$, so as to get a comparable latent encoding of $\mathbf{x}^B$, $\mathbf{z}_B$. This common density model encoder allows us to elucidate differences in the simulations not visible in the high dimensional dataspaces, $\mathbf{x}^A$ and $\mathbf{x}^B$.

We use the same three common physical metrics (Intensity, Turbulent Length Scale (TLS), Land Fraction) in all nine simulations. This consistency allows us to obtain a coherent understanding of convective organization across the various latent

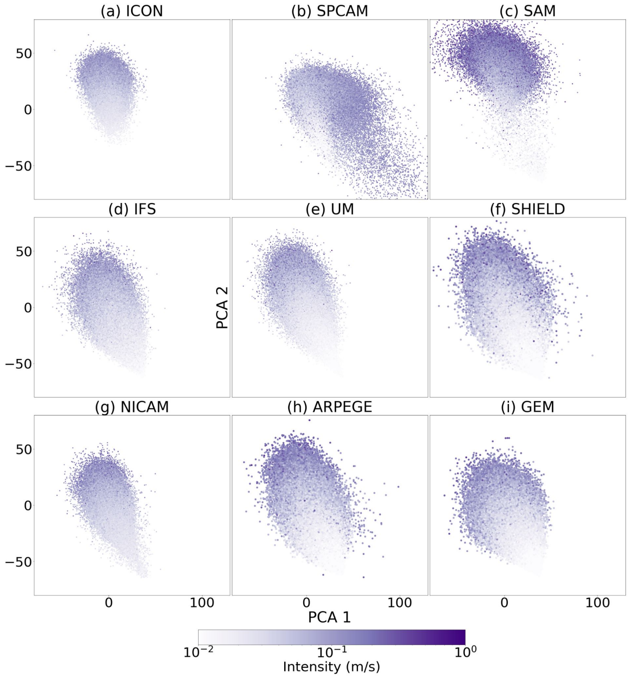

Fig. S5. Two-dimensional PCA plots of DYAMOND data encoded with a shared VAE (trained on UM data). Data points colorized by the mean of the absolute value of all updrafts in the vertical velocity field. We see a clear separation in the latent space of convection by the intensity of updraft (light purple vs. dark). SAM data (c) shows greater intensity (darker purples) compared to other DYAMOND GSRMs. Movie S4 shows a 3D visualization.

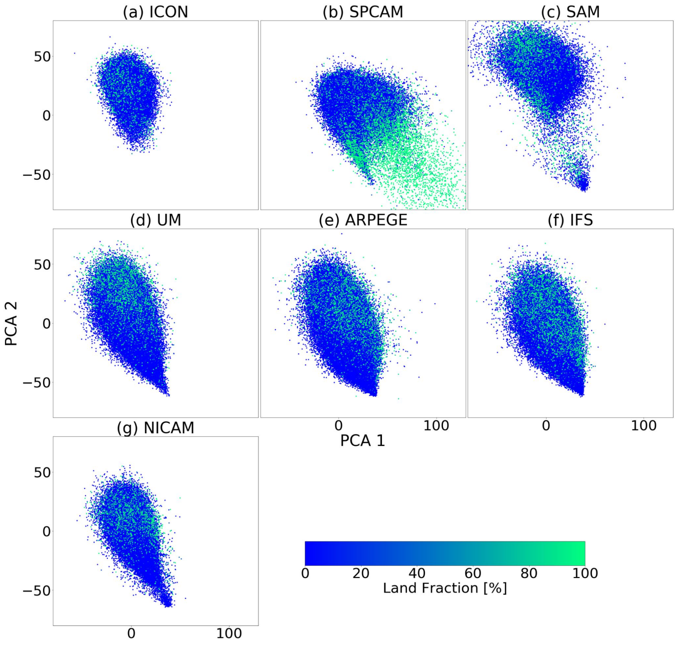

Fig. S6. Two-dimensional PCA plots of DYAMOND data encoded with a shared VAE (trained on UM data). Data points are colorized by the surface type (continent or ocean) of each vertical velocity field. We see disentanglement in the latent space between convection occurring over land and convection occurring over the ocean (green vs. blue). In SPCAM (b) we see a unique regime of a subsection of continental convection. GEM and SHIELD were left off due to missing land masks in the data. See Movie S5 for a full animation of the latent space in 3D.

representations generated by our encoder. While the level of disentanglement may vary across different test datasets and physical metrics, we observe consistent patterns across all latent spaces. This suggests that our approach possesses a degree of generalizability, enabling us to investigate high-dimensional GSRMs effectively.

F. Interpreting a latent space.

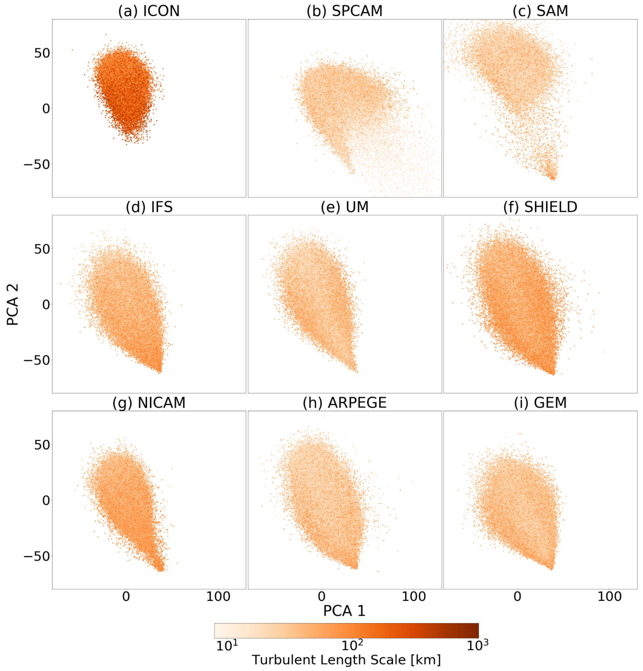

Fig. S7. Two-dimensional PCA plots of DYAMOND data encoded with a shared VAE (trained on UM data). Data points are colorized by the Turbulent Length Scale of each vertical velocity field (See Equation S3). The latent space separates out vertical velocity fields by the horizontal extent of convective updrafts (light orange vs. dark). This perspective reveals the unique land regime of convection in SPCAM (Figure S6b) to be defined by small-scale horizontal organization. See Movie S6 for a full animation of the latent space in 3D.

Visualization VAEs are known to reveal interpretable and disentangled properties in a lower-dimensional space. In our study, we investigate whether this principle holds for high-resolution convection by employing a VAE to encode DYAMOND GSRM simulation data. Through this approach, we aim to uncover interpretable structures and create visually rich representations in a low-dimensional space. Although we focus solely on data from the GSRM "UM" for the sake of brevity, it is important to note that this analysis applies to all nine simulation datasets.

In order to assess whether our latent space effectively *disentangles* the data based on meaningful criteria, we assign labels to each data point using widely accepted metrics that differentiate distinct types of convection, such as intensity and geography. Subsequently, we utilize two-dimensional Principle Component Analysis (PCA) projections of the latent space to visually represent this information. Specifically, we *colorize* the data points according to the chosen properties. Notably, the intensity measure exhibits a strong correlation with the y-axis ($R^2 > 0.6$), indicating that it primarily accounts for the main variation observed in the latent space. Additionally, we observe correlations on the x-axis with a metric representing the dominant turbulent horizontal length scale, providing insights into the width of vertical velocity updrafts. Interestingly, the x-axis also reveals geographic disentanglements, distinguishing between continental and maritime convection, despite the fact that geographic location or land-sea contrast was not included in the training data.

Clustering To formally evaluate the level of *disentanglement* in our latent space, we cluster the first couple principal components of the latent representations and examine the physical properties of each cluster. We choose a K-means clustering algorithm to identify distinct convection regimes. As shown in Figure 3 (d-h), when we set $K = 3$, we find three distinct clusters in GSRMs. We observe that each cluster has different intensity statistics* and is made up of convection from different tropical regions.

The first cluster, called "Continental Shallow" Convection, comes from shallow morning convection over drier land surfaces in tropical regions (Figure 4b). This cluster has a bottom-heavy vertical velocity variance profile, as defined by $\sqrt{\overline{w'w'}}$ based on Equation 1. The second cluster, called "Deep" Convection, captures intense tropical convection over warm ocean surfaces such as the Indian Ocean and West Pacific Warm pool (Figure 4c). This cluster has an especially top-heavy and intense vertical velocity variance profile. The third cluster, called "Marine Shallow" Convection, captures less intense convection and low clouds in the rest of the tropical ocean, particularly on the western coasts of subtropical latitudes (Figure 4d). This cluster has a low vertical velocity variance profile.

G. Additional specifics of the visualization of modes of convection via vertical velocity in GSRMs.

It is important to note that the way in which we analyze vertical velocity is different than much of the existing analysis. Traditionally, the vertical modes of the atmosphere used to distinguish different forms of convection are visualized with large scale variables including omega, zonal winds, and heating profiles (10, 15, 17, 18, 24). When plotted against pressure these coarse variables can highlight the baroclinic modes associated with deep convection (strong mid-tropospheric effects from latent heating through condensation)

*the summed absolute magnitude of vertical velocity across the input image

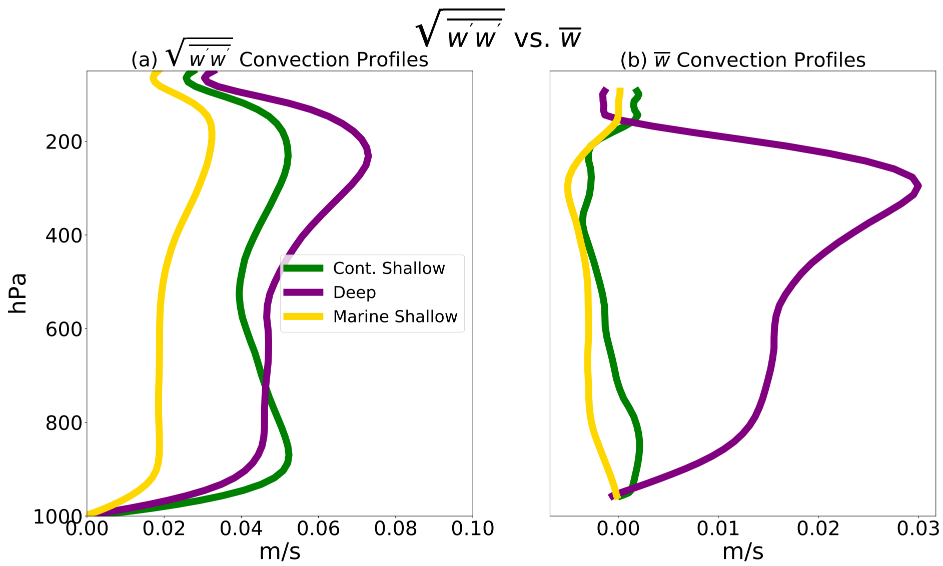

Fig. S8. A modified version of Figure 3 d, but showing the median cluster profiles in the form of the more traditional $\overline{w}$ (b) instead of how we visualize vertical velocity throughout the rest of the paper (a) as $\sqrt{\overline{w'w'}}$.

and stratiform convection (relatively consistent but less intense vertical motion) as well as a shallow mode (heating in the lower troposphere after cumulus formation) (10, 18). This approach has value in the analysis of tropical convection but is heavily dependent on closure assumptions and parameterizations (24).

Because we are working at storm-resolving scales, we use small scale vertical velocity to capture modes of convection, and more specifically hone in on the variance throughout the vertical profile via Equation 3. This approach captures three modes but in a different form than the traditional baroclinic modes. To help orient those unfamiliar with $\sqrt{\overline{w'w'}}$ profiles we modify Figure 4a to not only show $\sqrt{\overline{w'w'}}$ but more traditionally averaged $\overline{w}$ profiles in Figure S8. This comparison works well in our study because we are working on sub-domains where $\overline{w}$ is not 0. In Figure S8, when we look at the clusters from $\overline{w}$, we see modes lower in the troposphere in a more familiar baroclinic structure.

H. Additional details on Convection Types assigned by our Common VAE Encoder. Our procedure to uncover the physical characteristics of the convection in each cluster of the latent space is covered here in greater depth. While the visual representations of the latent space (Figures 3, S5- S7) provide valuable insights into the discrepancies among GSRMs, it is equally important to gain a deeper understanding of the specific nature and underlying reasons behind these differences. However, this task presents challenges due to the large volume of data involved, with each test dataset containing hundreds of thousands of vertical velocity fields, and the limitations of human perception at the native resolution of these fields.

However, we can still summarize this information in an interpretable manner. Firstly, we can perform an averaging process to collapse the horizontal dimension of all the vertical velocity fields in the test data. This reduces the 2D fields to their first moment statistics ($\sqrt{\overline{w'w'}}$ from Equation 3). Subsequently, within each cluster, we can average these $\sqrt{\overline{w'w'}}$ profiles together to get a representation of the typical vertical structure of each regime of convection (Figure S9).

We can use this approximation for both intra- and inter-GSRM comparisons based on the latent space's clustered convection types. Specifically, we can examine the extent to which different regimes of convection within a single GSRM have meaningfully different vertical profiles (Figure S9, a vs. b vs. c – different subplots but same color profiles). Furthermore, we can assess the similarity of the same species of convection across different GSRM simulation outputs (Figure S9, differences in vertical profiles in the same subplot). We acknowledge that there is some loss of information from neglecting the horizontal dimension as well as from the data extremes because we choose to visualize the mean. Nevertheless, this framework remains valuable for obtaining a composite perspective on the interpretability of these unsupervised convection regimes and the extent of their generalizability across different GSRMs.

Another issue arising from the relatively large test dataset sizes is the difficulty in visualizing density differences in the latent spaces using only two dimensions. To gain a better understanding of the nature of GSRM simulation outputs, we examine cluster proportions. While two GSRM simulations may appear to have the same three types of convection based on the results shown in Figures S5- S7, this does not necessarily imply that these convection regimes will occur at the same proportion in both simulations. In Figure S10, we present the quantification of the proportion of convection assigned to each cluster, enabling a more comprehensive comparison of these extensive test datasets. In the case of ICON (Figure S10, row 3), which exhibits vertical profiles and a latent representation similar to most other GSRMs (Figures S5- S7), Figure S10 demonstrates that the proportion of different convection species within ICON (as well as SPCAM and SAM) differs from that in other GSRMs.

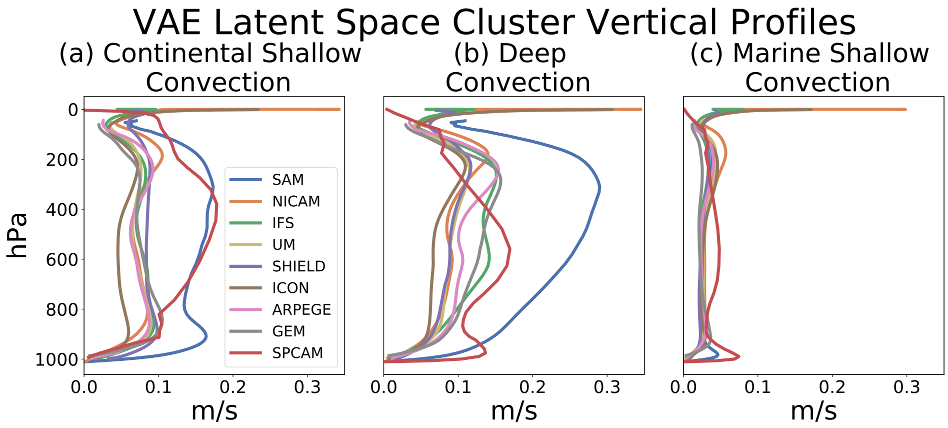

Fig. S9. The mean $\sqrt{\overline{w'w'}}$ (Equation 3) profile of each cluster of convection across all nine GSRM simulation outputs. The centroids used to organize the other eight GSRM simulations are fixed by initial clustering on the UM latent space. Overall, we see common types of convection identified across GSRMs (similar vertical velocity fields clustered in the same parts of the latent space regardless of input data type). SAM (blue curves) and SPCAM (red curves) stand out as unique from the typical vertical structure of a GSRM convection regime.

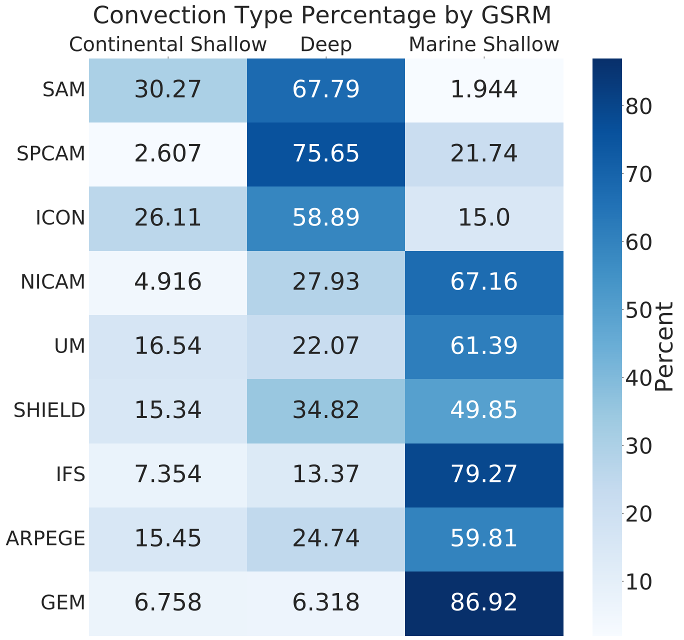

Fig. S10. The proportion of vertical velocity fields assigned to each of the three regimes of convection across the nine simulations. As in Figure S9, the centers initialized in $\mathbf{z}_{UM}$ are used to assign labels to data in across all nine simulations. We see a split across the DYAMOND simulations (top three rows vs. all). SAM, SPCAM, and ICON all assign much high proportions of convection in their test datasets to the more intense regimes compared to other DYAMOND GSRMs.

I. On the Nature of Convection Types in SPCAM. We examine the proportion of each type of convection at every latitude-longitude grid-cell (Figure S11 for our +4K SPCAM simulation; not shown for control climate). This analysis yields three physically distinct and interpretable geographic patterns of convection.

These three convective species, organized by the latent space of our VAE, provide for a clean comparison with previous literature on tropical meteorology which also typically identifies three distinct convection types (10, 17, 24). Both approaches isolate a cluster of “Deep Convection” (Figure S11b). However, historically, the remainder of tropical convection, visualized from the two baroclinic modes of vertical velocity or other summary statistics (cloud top height, precipitation or maximum updraft intensity), is classified as cumulus congestus (or stratiform) and shallow cumulus (12, 16, 20). Our VAE latent space unites these two groups into one regime, which we call simply “Shallow Convection”, while simultaneously isolating an unusual “Continental Shallow Cumulus” mode of convection (Figure S11 a and c).

In summary, our findings suggest that the organization of tropical convection through unsupervised methods will yield different patterns compared to those found in traditional physically informed approaches. However, both methods deliver results that align with domain knowledge, highlighting how unsupervised machine learning models can complement and even augment traditional analysis.

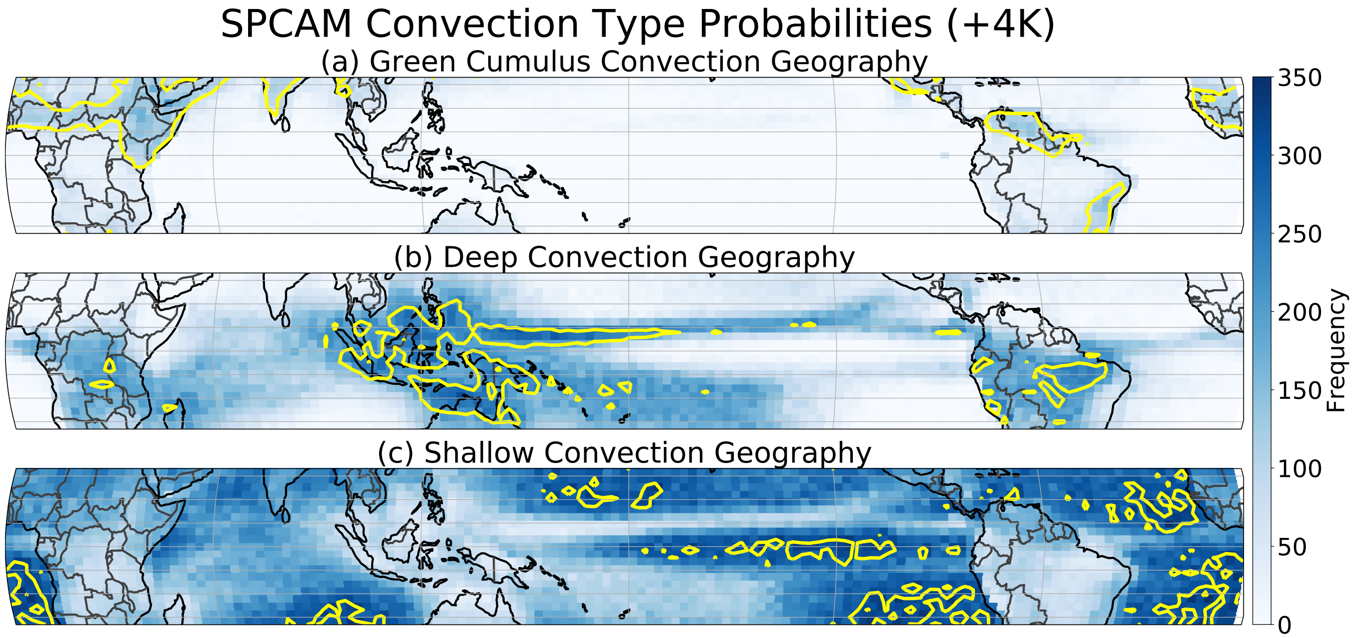

Fig. S11. The geographic domain of each of the three regimes of convection organized by the VAE latent space in SPCAM. More specifically, we total the number of instances of a regime of convection identified at each lat/lon grid cell. Results are shown for SPCAM +4K data (Not shown for the 0K control climate but findings are similar). Yellow contour lines encompass the 92.5 percentile for each regime. Though not identical to the convective species typically identified by physically informed approaches, these convection types found by the VAE all have distinct physical properties and geographic extents which would justify their separation from a domain perspective.

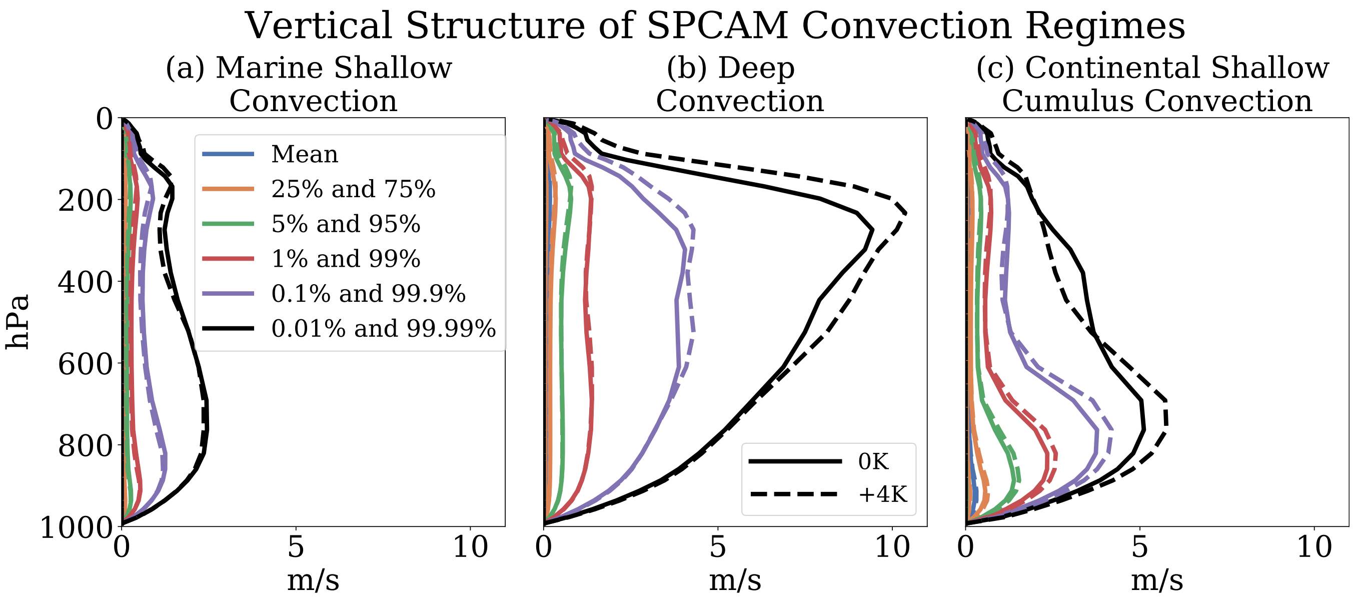

Fig. S12. A comprehensive view of the vertical structure of each type of convection in SPCAM and how it changes as temperatures rise (solid vs. dashed lines). But instead of only restricting ourselves to a view of the mean, we look at percentiles across the test data in each convection cluster. The VAE anticipates both an increase in the most intense deep convection with warming (b) and a strengthening of turbulent updrafts in the boundary layer (c).

J. Expanded analysis of Convection Cluster shifts with warming. The effects of climate change often become more evident in extreme events, necessitating an analysis beyond the mean values of GSRM simulation data and considering the tails of the probability density functions (PDFs). This expanded analysis is particularly valuable when examining the vertical structure of convection. If we only focused on the means (Figure 6d), we would conclude that convective intensity systematically decreases with global warming. However, by also examining the profiles of extreme vertical velocity fields, we can observe that the most intense convective structures within the Deep Convection regime (above the 99th percentile) actually intensify with climate change (Figure S12b). Moreover, this analysis reveals the previously concealed signal of intensified boundary layer turbulence in arid continental zones (Figure S12c).

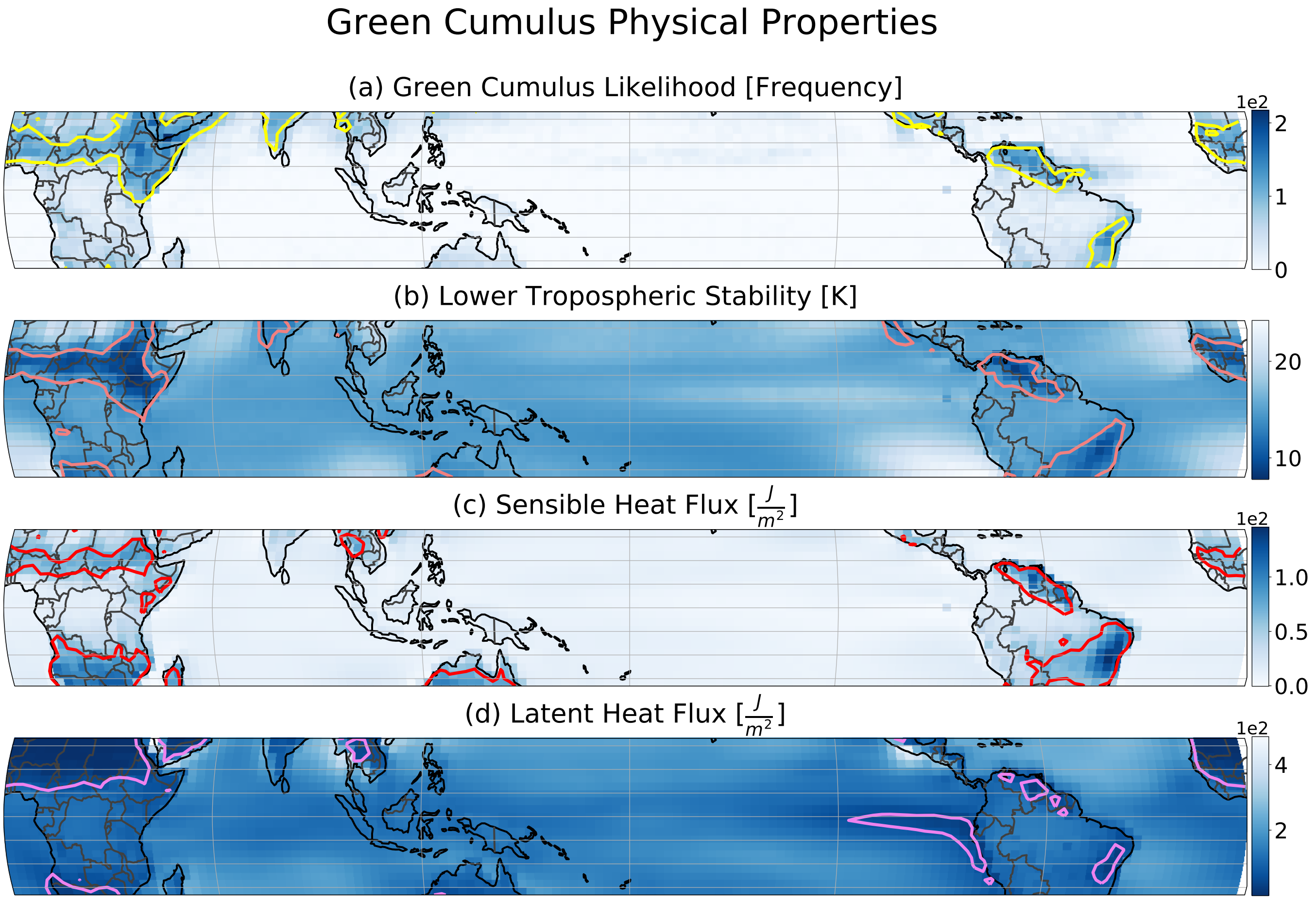

Fig. S13. We identify the atmospheric conditions that enable the growth and development of “Continental Shallow Cumulus” (or “Green Cumulus”). The regions where “Green Cumulus” convection occurs most frequently (a) are contoured against the patterns of various physical measures of atmospheric conditions (b,c,d). We find “Green Cumulus” can be classified by small Lower Tropospheric Stability (b), large Sensible Heat Flux (c), and low Latent Heat Flux (d). Contours cover the 92.5 percentile (a,c) and the 7.5 percentile (b,d).

K. Green Cumulus Convection. We expand on efforts to diagnose the physical properties that compose “Green Cumulus" convection. Historically, it has been challenging to establish “Green Cumulus" as a separate category due to its infrequent occurrence and similarities in physical attributes such as potential temperature, specific humidity, relative humidity, and large-scale omega compared to other well-established types of convection (7). But through both analysis of its vertical structure, as defined by small-scale vertical velocity (Figure S12c), and by examining its associated surface fluxes (Figure S13), we can better quantify this mode of convection. It is defined by intense updrafts in the boundary layer which are far larger than any other mode of convection. However, in the upper troposphere, we find very weak updrafts during periods of “Green Cumulus" (Figure S12c). Conditions for the growth of “Green Cumuli” are most favorable when Lower Troposphere Stability is small, Sensible Heat Flux is high, and Latent Heat Flux is relatively low (Figure S13). This suggests “Green Cumulus” is the dominant regime over land regions where conditions are semi-dry but not extremely dry like deserts.

We believe our efforts to better understand “Green Cumulus” can yield benefits for atmospheric modeling and physical process understanding. While our VAE identifying Green Cumulus is not a novel discovery, it is valuable to investigate because it is an understudied form of convection (1, 6, 26) compared to the marine variant which is much easier to simulate given its lack of a diurnal cycle and weaker surface fluxes allowing for an assumption of quasi-equilibrium when modeled (28). While some campaigns like AmazonGO and ARM have provided observational data and a basis for some simulation of this “Green Cumulus” (8, 27), these studies have been restricted geographically (to just the Southern Great Plains and the Amazon Basin). Analysis using satellite data offers a spatially richer view but lower temporal resolution (5, 6). The study of this convective regime is further limited because it is missing in the GOES ABI cloud mask (23). Our VAE extracts this unique “Green Cumulus” mode regardless of its geographic domain in large SPCAM simulations with high temporal frequency (15 minute time-step). This can improve our understanding of “Green Cumuli” behavior with respect to both short temporal transitions and full seasonal timescales, which are currently lacking to due sampling limitations and inconsistencies (8, 29). This physical understanding is crucial because this mode of convection has a typical domain size on the order of just one kilometer (14, 26) necessitating its parameterization in models. Improvements in physical understanding of this “Green Cumulus" through more rigorous spatial and temporal analysis could help build superior parameterizations, potentially resolving downstream problems

stemming from unconstrained shallow cloud representation, including premature shallow-to-deep convection transition and associated temporal precipitation inaccuracies in current climate simulations (3, 11, 25).

Movie S1. 250 examples of vertical velocity snapshots used as training data from each of the nine GSRMs we examine in the scope of this paper. We observe a variety of convection formations and species. The movie can be viewed at the link here.

Movie S2. Three-dimensional PCA animation of UM Data encoded by a VAE. Data points are colorized by physical convection properties, including convection intensity (a), land fraction (b), turbulent length scale (c), and by convection type (as found by clustering) (d). We see evidence of disentanglement by all four metrics. For a different visual perspective, we increase transparency to 99.9 % (a,b,d) or 99 % (c) to better show the latent representation of the full test dataset (size 125,000). The movie can be viewed at the link here.

Movie S3. Three-dimensional PCA animation of DYAMOND data encoded with a shared VAE (trained on UM data). Latent data is colorized by convection type (as found by clustering). The top panels (b and c) show clear differences in their latent organization compared to the remaining models. The movie can be viewed at the link here.

Movie S4. Three-dimensional PCA animation of DYAMOND data encoded with a shared VAE (trained on UM data). Latent data is colorized by the mean of the absolute intensity of the vertical velocity field. The latent representation of SAM (c) shows much greater intensities than other GSRMs. The movie can be viewed at the link here.

Movie S5. Three-dimensional PCA animation of DYAMOND data encoded with a shared VAE (trained on UM data). Latent data is colorized by the surface type (land or ocean) of the vertical velocity field. In the latent representation of SPCAM (b) we see a unique regime of continental shallow convection (green). GEM and SHIELD left off due to missing land masks in data. The movie can be viewed at the link here.

Movie S6. Three-dimensional PCA animation of DYAMOND data encoded with a shared VAE (trained on UM data). Latent data is colorized by the Turbulent Length Scale of each vertical velocity field (See Equation S3). The latent space separates vertical velocity fields by the horizontal extent of convective updrafts (light orange vs. dark). This perspective reveals the unique land regime of convection in SPCAM (Movie S5) to be defined by small-scale horizontal organization. The movie can be viewed at the link here.

References

1. Maike Ahlgrimm and Richard Forbes. The impact of low clouds on surface shortwave radiation in the ecmwf model. *Monthly Weather Review*, 140(11):3783 – 3794, 2012. . URL https://journals.ametsoc.org/view/journals/mwre/140/11/mwr-d-11-00316.1.xml.
2. David Arthur and Sergei Vassilvitskii. K-means++: The advantages of careful seeding. In *Proceedings of the Eighteenth Annual ACM-SIAM Symposium on Discrete Algorithms*, SODA '07, page 1027–1035, USA, 2007. Society for Industrial and Applied Mathematics. ISBN 9780898716245.
3. P. Bechtold, J.-P. Chaboureau, A. Beljaars, A. K. Betts, M. Köhler, M. Miller, and J.-L. Redelsperger. The simulation of the diurnal cycle of convective precipitation over land in a global model. *Quarterly Journal of the Royal Meteorological Society*, 130(604):3119–3137, 2004. . URL https://rmets.onlinelibrary.wiley.com/doi/abs/10.1256/qj.03.103.
4. Raymond B. Cattell. The scree test for the number of factors. *Multivariate Behavioral Research*, 1(2):245–276, 1966. . URL https://doi.org/10.1207/s15327906mbr0102_10. PMID: 26828106.
5. T. Dror, M. D. Chekroun, O. Altaratz, and I. Koren. Deciphering organization of goes-16 green cumulus through the empirical orthogonal function (eof) lens. *Atmospheric Chemistry and Physics*, 21(16):12261–12272, 2021. . URL https://acp.copernicus.org/articles/21/12261/2021/.
6. Tom Dror, Ilan Koren, Orit Altaratz, and Reuven H. Heiblum. On the abundance and common properties of continental, organized shallow (green) clouds. *IEEE Transactions on Geoscience and Remote Sensing*, 59(6):4570–4578, 2021. .
7. Tom Dror, Vered Silverman, Orit Altaratz, Mickaël D. Chekroun, and Ilan Koren. Uncovering the large-scale meteorology that drives continental, shallow, green cumulus through supervised classification. *Geophysical Research Letters*, 49 (8):e2021GL096684, 2022. . URL https://agupubs.onlinelibrary.wiley.com/doi/abs/10.1029/2021GL096684. e2021GL096684 2021GL096684.
8. Alice Henkes, Gilberto Fisch, Luiz A. T. Machado, and Jean-Pierre Chaboureau. Morning boundary layer conditions for shallow to deep convective cloud evolution during the dry season in the central amazon. *Atmospheric Chemistry and Physics*, 21(17):13207–13225, sep 2021. . URL https://doi.org/10.5194%2Facp-21-13207-2021.

9. Sakshi Indolia, Anil Kumar Goswami, S.P. Mishra, and Pooja Asopa. Conceptual understanding of convolutional neural network- a deep learning approach. *Procedia Computer Science*, 132:679–688, 2018. ISSN 1877-0509. . URL https://www.sciencedirect.com/science/article/pii/S1877050918308019. International Conference on Computational Intelligence and Data Science.
10. Richard H. Johnson, Thomas M. Rickenbach, Steven A. Rutledge, Paul E. Ciesielski, and Wayne H. Schubert. Trimodal characteristics of tropical convection. *Journal of Climate*, 12(8):2397 – 2418, 1999. . URL https://journals.ametsoc.org/view/journals/clim/12/8/1520-0442_1999_012_2397_tcotc_2.0.co_2.xml.
11. Marat Khairoutdinov and David Randall. High-resolution simulation of shallow-to-deep convection transition over land. *Journal of the Atmospheric Sciences*, 63(12):3421 – 3436, 2006. . URL https://journals.ametsoc.org/view/journals/atsc/63/12/jas3810.1.xml.
12. Boualem Khouider and Andrew J. Majda. A simple multicloud parameterization for convectively coupled tropical waves. part i: Linear analysis. *Journal of the Atmospheric Sciences*, 63(4):1308 – 1323, 2006. . URL https://journals.ametsoc.org/view/journals/atsc/63/4/jas3677.1.xml.
13. Alex Krizhevsky, Ilya Sutskever, and Geoffrey E Hinton. Imagenet classification with deep convolutional neural networks. *Advances in neural information processing systems*, 25, 2012.
14. Katia Lamer and Pavlos Kollias. Observations of fair-weather cumuli over land: Dynamical factors controlling cloud size and cover. *Geophysical Research Letters*, 42(20):8693–8701, 2015. . URL https://agupubs.onlinelibrary.wiley.com/doi/abs/10.1002/2015GL064534.
15. Andrew J. Majda and Michael G. Shefter. Models for stratiform instability and convectively coupled waves. *Journal of the Atmospheric Sciences*, 58(12):1567 – 1584, 2001. . URL https://journals.ametsoc.org/view/journals/atsc/58/12/1520-0469_2001_058_1567_mfsiac_2.0.co_2.xml.
16. Andrew J. Majda, Boualem Khouider, George N. Kiladis, Katherine H. Straub, and Michael G. Shefter. A model for convectively coupled tropical waves: Nonlinearity, rotation, and comparison with observations. *Journal of the Atmospheric Sciences*, 61(17):2188 – 2205, 2004. . URL https://journals.ametsoc.org/view/journals/atsc/61/17/1520-0469_2004_061_2188_amfcct_2.0.co_2.xml.
17. Brian E. Mapes. Convective inhibition, subgrid-scale triggering energy, and stratiform instability in a toy tropical wave model. *Journal of the Atmospheric Sciences*, 57(10):1515 – 1535, 2000. . URL https://journals.ametsoc.org/view/journals/atsc/57/10/1520-0469_2000_057_1515_cisste_2.0.co_2.xml.
18. Hirohiko Masunaga and Tristan S. L'Ecuyer. A mechanism of tropical convection inferred from observed variability in the moist static energy budget. *Journal of the Atmospheric Sciences*, 71(10):3747 – 3766, 2014. . URL https://journals.ametsoc.org/view/journals/atsc/71/10/jas-d-14-0015.1.xml.
19. Griffin Mooers, Jens Tuyls, Stephan Mandt, Mike Pritchard, and Tom G Beucler. Generative modeling of atmospheric convection. In *Proceedings of the 10th International Conference on Climate Informatics*, CI2020, page 98–105, New York, NY, USA, 2020. Association for Computing Machinery. ISBN 9781450388481. . URL https://doi.org/10.1145/3429309.3429324.
20. Matthew E. Peters and Christopher S. Bretherton. Structure of tropical variability from a vertical mode perspective. *Theoretical and Computational Fluid Dynamics*, 20(5):501–524, 2006.
21. Evgeny A Smirnov, Denis M Timoshenko, and Serge N Andrianov. Comparison of regularization methods for imagenet classification with deep convolutional neural networks. *Aasri Procedia*, 6:89–94, 2014.
22. Christian Szegedy, Alexander Toshev, and Dumitru Erhan. Deep neural networks for object detection. *Advances in neural information processing systems*, 26, 2013.
23. Yang Tian, Yunyan Zhang, Stephen A. Klein, and Courtney Schumacher. Interpreting the diurnal cycle of clouds and precipitation in the arm goamazon observations: Shallow to deep convection transition. *Journal of Geophysical Research: Atmospheres*, 126(5):e2020JD033766, 2021. . URL https://agupubs.onlinelibrary.wiley.com/doi/abs/10.1029/2020JD033766. e2020JD033766 2020JD033766.
24. Stefan N. Tulich, David A. Randall, and Brian E. Mapes. Vertical-mode and cloud decomposition of large-scale convectively coupled gravity waves in a two-dimensional cloud-resolving model. *Journal of the Atmospheric Sciences*, 64(4):1210 – 1229, 2007. . URL https://journals.ametsoc.org/view/journals/atsc/64/4/jas3884.1.xml.
25. Jun Yin and Amilcare Porporato. Diurnal cloud cycle biases in climate models. *Nature Communications*, 8(1):2269, 2017. . URL https://doi.org/10.1038/s41467-017-02369-4.
26. Yunyan Zhang and Stephen A. Klein. Factors controlling the vertical extent of fair-weather shallow cumulus clouds over land: Investigation of diurnal-cycle observations collected at the arm southern great plains site. *Journal of the Atmospheric Sciences*, 70(4):1297 – 1315, 2013. . URL https://journals.ametsoc.org/view/journals/atsc/70/4/jas-d-12-0131.1.xml.
27. Yunyan Zhang, Stephen A. Klein, Jiwen Fan, Arunchandra S. Chandra, Pavlos Kollias, Shaocheng Xie, and Shuaiqi Tang. Large-eddy simulation of shallow cumulus over land: A composite case based on arm long-term observations at its southern great plains site. *Journal of the Atmospheric Sciences*, 74(10):3229 – 3251, 2017. . URL https://journals.ametsoc.org/view/journals/atsc/74/10/jas-d-16-0317.1.xml.
28. Ping Zhu and Bruce Albrecht. Large eddy simulations of continental shallow cumulus convection. *Journal of Geophysical Research: Atmospheres*, 108(D15), 2003. . URL https://agupubs.onlinelibrary.wiley.com/doi/abs/10.1029/2002JD003119.
29. Yizhou Zhuang, Rong Fu, José A. Marengo, and Hongqing Wang. Seasonal variation of shallow-to-deep convection transition and its link to the environmental conditions over the central amazon. *Journal of Geophysical Research: Atmospheres*, 122(5):2649–2666, 2017. . URL https://agupubs.onlinelibrary.wiley.com/doi/abs/10.1002/2016JD025993.
